# ‘Heroes Were (And Could Be) Created’: An Exploration of Adolescents' Perspectives of Portrayals of Pain and Empathy in Media

**DOI:** 10.1002/ejp.70315

**Published:** 2026-06-17

**Authors:** Allison Cormier, Sean Lindsay, Samantha Noyek, Katya Dittrich, Abbie Jordan, Kathryn A. Birnie, Melanie Noel

**Affiliations:** ^1^ Werklund School of Education, School and Applied Child Psychology, University of Calgary Calgary Alberta Canada; ^2^ Department of Psychology University of Calgary Calgary Alberta Canada; ^3^ Department of Pediatrics McMaster University, Health Sciences Centre Hamilton Ontario Canada; ^4^ Department of Psychology University of Bath Bath UK; ^5^ Centre for Pain Research, University of Bath Bath UK; ^6^ Department of Anesthesiology, Perioperative and Pain Medicine University of Calgary, Foothills Medical Centre Calgary Alberta Canada; ^7^ Department of Community Health Sciences University of Calgary Calgary Alberta Canada; ^8^ Alberta Children's Hospital Research Institute Calgary Alberta Canada; ^9^ Hotchkiss Brain Institute, University of Calgary, Health Research Innovation Centre Calgary Alberta Canada

## Abstract

**Background:**

Research suggests that media consumption plays a role in socialization, including the socialization of painful experiences. Findings from previous research examining pain depictions in popular adolescent media suggest that violence and injuries are frequently represented, marginalized populations are underrepresented, and observers lack prosocial responses to sufferers' pain. This study was the first to examine how adolescents perceive, internalize, and discuss pain depictions in popular adolescent media.

**Methods:**

Fourteen adolescents (between 13 and 18 years of age), both with and without chronic pain, participated in focus groups. Participants watched clips depicting pain in popular adolescent media before providing their feedback.

**Results:**

Using reflexive thematic analysis, three themes were generated: (1) a failure to communicate, but not to empathize—observers may be fearful or lack the tools and confidence to address sufferer pain, (2) media as the metric—media serves as the metric by which we can compare pain experiences, which may contribute to stigma and disbelief, and (3) media as the conduit for transformation—media may be harnessed to properly represent and build empathy for pain.

**Significance:**

These novel findings suggest that media portrayals of pain may shape youths' beliefs about pain expression, validation, and empathy. Media could therefore serve as a modifiable target for reducing pain‐related stigma and improving empathic responses to pain.

## 1 Introduction

Acute pain (e.g., injuries, medical procedures) affects all adolescents and 20% will experience chronic pain (Chambers et al. 2024). Despite this high prevalence, paediatric pain is often undertreated (De Ruddere and Craig 2016) and parents do not often engage their children in validating conversations about pain (Pavlova et al. 2022). Consequently, adolescents may learn about pain from other sources including their own pain experiences, observing others' pain, and societal influences including media (Paek et al. 2011).

Media provides adolescents with opportunities to learn through observation (Bandura 1986). High exposure to violent media is linked to desensitization and reduced empathy, while exposure to prosocial depictions can foster positive behaviours (Carnagey et al. 2007; Prot et al. 2015). Similarly, media shapes stereotypes and societal attitudes; even minimal exposure influences perceptions of marginalized groups (Dixon 2006; Entman and Rojecki 2000). Given the prominence of media in adolescents' lives (Madigan et al. 2022), its portrayal of pain warrants examination.

Previous research indicates that pain is frequently depicted in adolescent television series and movies, occurring 10.24 times/h (Cormier et al. 2024), providing adolescents with ample learning opportunities about pain. These depictions overwhelmingly focus on violent pain (77%), with chronic‐type pains and medical/procedural pains accounting for fewer than 2% of instances (Cormier et al. 2024). Researchers argue that the underrepresentation of chronic‐type pains and medical/procedural pains may be a missed opportunity for adaptive learning (Cormier et al. 2024; Mueri et al. 2021). Additionally, when characters experience pain, they generally have neutral responses to pain and rarely engage in protective behaviours, while observers frequently respond maladaptively or fail to show empathy (Cormier et al. 2024). This lack of prosocial behaviours may suggest that pain is unworthy of care and must be endured in isolation, possibly reinforcing stigmatizing attitudes.

Media depictions of pain are influenced by gender and racial identity. Despite girls reporting more pain than boys (King et al. 2011), they are underrepresented as people with lived experience in the media (23%). Racialized people are also underrepresented (22% vs. 78%) and racialized characters are more likely to experience pain provoked by others, perhaps reflecting systemic oppression (Cormier et al. 2024; Green et al. 2003). Pain instances were more commonly experienced by boy characters, and this pain was rated as being more severe; yet observers were more prosocial towards girl characters' pain and more likely to mock/taunt boy characters' pain. When a racialized character experienced pain, they were more likely to experience pain caused by another character. Their pain was underrepresented, but when it was depicted, they were more likely to have observers both victimize and empathize with them (Cormier et al. 2024). These patterns perpetuate harmful stereotypes and may lead marginalized groups to internalize beliefs that their pain is less valid (Mueri et al. 2021).

Research has established the importance of media in adolescents' socialization and has characterized pain depictions they consume. The current study extended this work by exploring how adolescents perceive and discuss portrayals of pain and empathy in popular media.

## 2 Methodology

### 2.1 Study Design

This exploratory, qualitative study explored adolescents' perceptions of pain in popular adolescent media. Three focus groups were conducted via Zoom from November 2022 to January 2023. Each focus group met one time. Two focus groups were held for adolescents without chronic pain. These groups were each composed of four participants. To ensure representation of the lived experience of adolescent pain (given research showing that media depictions overwhelmingly focus on violent pain, with chronic‐type pains accounting for fewer than 2% of instances; Cormier et al. 2024), an additional focus group was held for six adolescents with chronic pain. This was not intended to draw conclusions about differences in perceptions of youth with and without chronic pain, but rather to ensure that there was diversity in our participant sample regarding lived experiences of pain. Focus groups were conducted separately for adolescents with versus without chronic pain to promote comfortable discussion among participants and alignment based on that aspect of lived experience.

The limited number of focus groups included in this study was both a pragmatic decision, stemming from the challenges of recruiting youth with lived experience of pain, and a methodological choice consistent with reflexive thematic analysis (Braun and Clarke 2021). While a larger sample might have yielded additional perspectives, our emphasis was on creating space for engagement and reflexivity, both in the open and conversational focus groups, and in the interpretation of the data.

A focus group schedule (see Appendix S1) was informed by a patient partner and co‐author (K.D.) and considered previous research characterizing pain in popular adolescent media (Cormier et al. 2024). Participants were asked to provide their feedback about what they noticed regarding the role of race and/or gender, the realism of pain depictions, observer responses to pain, and what should change about pain depictions in popular media. The patient partner has lived experience with paediatric chronic pain and was compensated as per best practices to reflect her knowledge and expertise (Richards et al. 2018). K.D. has previously been a patient partner for several studies led by the primary investigator (M.N.). The patient partner co‐facilitated the first focus group to inform how all subsequent focus groups would proceed.

### 2.2 Materials

During the focus group, adolescents were presented with a reel of eight clips depicting pain in popular adolescent media (08:48 min total). The media series, length, and a brief description of each clip are provided in Table 1. Clips were purposively selected to provide a comprehensive snapshot of pain portrayals in adolescent media, guided by prior research on this topic (Cormier et al. 2024). Cormier et al. (2024) analysed a cross‐section of ten movies and six television shows featuring adolescent protagonists, drawn from Netflix trending/popular lists. Building on this foundation, researchers in the current study selected clips from the same cross‐section to ensure consistency and relevance. The first author (A.C.) identified the clips, which were then reviewed by the senior author (M.N.) to ensure a breadth of pain depictions.

**TABLE 1:** Pain depictions in popular adolescent media viewed by participants.

Name of television show	Length of clip	Description
Outerbanks	01:30 min	Two adolescent males attack a male with a golf club
Sex Education	30 s	Adolescent slams door into the nose of a female abortion protester
Outerbanks	02:20 min	Adolescent female is stung by a jellyfish
Sex Education	50 s	Male adolescent dressed in drag is physically assaulted by a bystander
13 Reasons Why	01:20 min	Male is pushed off the booth at a restaurant when he attempts to sexually assault a woman
Riverdale	15 s	Male character burns his hand on a hot pizza dish
Outerbanks	01:57 min	Male adolescent is injured and receiving first‐aid from a female bystander
13 Reasons Why	01:26 min	Male adolescent is experiencing chronic stomach pains

The final selection of clips included a representation of all types of pain (i.e., medical/procedural, every day, chronic‐type, violent, and injuries), with an emphasis placed upon the preponderance of violence and injuries commonly depicted in adolescent media. There was a breadth of observer responses to adolescents with lived experience of pain (AWLEP); both empathetic and non‐empathetic responses were included. Further, clips were selected to reflect the intersection of pain and racial identity, gender, and/or sexuality. The array of clips presented afforded participants the ability to discuss and comment on their perceptions of pain in popular adolescent media.

### 2.3 Participants

Participants included 14 adolescents between 13 and 18 years of age (*M* = 16.78, SD = 1.53) who either lived with (*n* = 6) or without (*n* = 8) chronic pain. Those with chronic pain most frequently reported pain in their muscles and joints (83%), head (33%), or other locations (33%) (i.e., feet, spine). Participant demographics are presented in Table 2. Adolescents were asked ‘what is your current gender identity’ and selected from the following options: male, female, trans male/man, trans female/woman, genderqueer/gender non‐conforming, or the option to identify a different gender identity.

**TABLE 2:** Sociodemographic characteristics of participants.

Characteristic	Group			
	Chronic pain		Non‐chronic pain	
	*N*	%	*N*	%
Gender				
Female	5	83	2	25
Male	1	17	5	62.5
Genderqueer	0	0	1	13.5
Race/Ethnicity				
Arab/West Asian	0	0	1	12.5
Black	0	0	1	12.5
Chinese	0	0	1	12.5
Southeast Asian	1	12.5	0	0
Mixed	0	0	1	12.5
White	5	83	4	50
Sexual Orientation				
Straight	5	83	5	62.5
Lesbian	0	0	1	12.5
Bisexual	0	0	2	25
Asexual	0	0	1	12.5
Pansexual	0	0	1	12.5
Questioning	1	12.5	0	0

*Note:* Participants were able to select more than one option to best reflect their identities.

Most adolescents in the chronic pain group identified as female, whereas one participant identified as male. Conversely, most of the non‐chronic pain group identified as male. Two participants identified as female, while another identified as genderqueer. In the chronic pain group, most participants self‐identified as white (83%). In the non‐chronic pain group, 64% of participants self‐identified as white, whereas 36% of participants self‐identified as BIPOC. All participants were required to speak and understand English and have access to the internet given that all focus groups were conducted via Zoom.

### 2.4 Procedure

Ethics approval was granted by the University of Calgary's Conjoint Faculties Research Ethics Board, University of Calgary (Ethics ID: REB21‐1308). One participant completed an assent form, accompanied by a parent who provided written informed consent, while all others gave written informed consent before enrolling in the study. Several recruitment strategies were employed including sharing the study advertisement via social media and contacting participants from our previous studies who provided consent to being contacted for study participation. Previous research has identified that marginalized groups are underrepresented in popular adolescent media (Cormier et al. 2024); therefore, individuals identifying as LGBTQ2S+ or BIPOC were strongly encouraged to participate, and this was highlighted in the recruitment advertisement. Participants met with the primary researcher via telephone to establish consent prior to attending the focus groups. Subsequently, participants completed an online questionnaire assessing socio‐demographic characteristics and their pain experiences. Virtual focus groups were conducted via Zoom and were composed of 4–6 participants.

Prior to beginning the focus groups, the moderator provided context about the purpose of the study. Participants were briefed on findings from previous research characterizing pain in popular adolescent media including (1) the lack of empathic and prosocial responses displayed by observers, (2) the preponderance of violence and injuries and (3) the underrepresentation of BIPOC and female pain. Participants were shown eight clips depicting pain instances in popular adolescent media. The moderator probed participants about what they noticed regarding the role of race and/or gender, the realism of pain depictions, observer responses to pain, and what should change about pain depictions in popular media. Focus groups were audio and video recorded and subsequently transcribed. Focus groups lasted between 58 and 82 min (*M* = 67.59, SD = 13.01). All names and identifiable information (e.g., geographical location) were removed from the transcripts for anonymity. Each participant received a $20 CAD gift card of their choice to Amazon, Indigo, or iTunes for their participation.

### 2.5 Data Analysis

Data was analysed using reflexive thematic analysis, which supports systematic analysis of meaning from participants' accounts (Braun and Clarke 2006). The analysis was comprised of the following steps: (1) the primary researcher transcribed all focus groups verbatim, grounding herself in the data; (2) familiarization with the data by carefully listening to and reading through each transcript; (3) initial codes were created to capture participants' ideas; (4) related codes were clustered to create a tentative interpretation of wider themes; (5) themes were compared across interviews to create a ‘thematic map’ of the data; (6) themes were further refined and named; and (7) a final list of themes was established. NVivo software (Lumivero 2021) was used to create initial codes and themes.

### 2.6 Quality in Qualitative Research

To achieve a high‐quality analysis, the current study incorporated the Cochrane Supplemental Guidance for Qualitative Evidence Synthesis and Shenton's (2004) strategies for ensuring trustworthiness. Credibility refers to the accuracy and fair representations of the phenomena under investigation (Hannes 2011). To achieve credibility, a patient partner was integrally involved in constructing the focus group schedule, ensuring that the questions posed were clear and relevant to the lived experiences of adolescents. The first author also conducted each focus group and transcribed them verbatim before proceeding to data analysis. This process allowed her to be deeply immersed in the data prior to drawing interpretations. Confirmability refers to the extent to which the results are grounded in the data (Hannes 2011). This was achieved by using NVivo (Lumivero 2021) to systematically link the data directly to emerging themes. Themes were cross‐checked with co‐authors to ensure that they were reflective of the data. Participant quotations were then extracted from the full range of participants and included throughout the results. Transferability (i.e., the extent the research findings can be applied outside of the participant sample) and dependability (i.e., the clarity of the research process) were achieved by providing appropriate context about the selection of media, sample demographics, and data analysis (Braun and Clarke 2006; Hannes 2011).

Finally, reflexivity about the active role of the researcher is a critical component of reflexive thematic analysis (Braun and Clarke 2006). The first author attended regular meetings with members of the research team (S.N. and M.N.) to discuss the analysis. Emerging themes were discussed and finalized in collaboration with the research team. A co‐author (S.L.), who was not involved in drawing the initial themes, reviewed the focus group transcripts to scan for the accuracy and completeness of data interpretations. Additionally, the patient partner (K.D.) reviewed the proposed themes and their associated quotes. The research team considered their positionality in relation to the participants and the data (Braun and Clarke 2006). AC primarily interacted with participants and is a white woman in her mid‐twenties with no visible disabilities. All co‐authors have a background in the study of adolescent chronic pain.

## 3 Results

Three themes were generated from the thematic analysis of the data: (1) ‘a failure to communicate empathy, not a failure to empathize’; (2) ‘media as the metric’; and (3) ‘media as the conduit for transformation’. These themes are presented with supporting verbatim quotes from adolescents, and it is specified whether they had lived with or without chronic pain. Pseudonyms are used for each participant to ensure confidentiality.

### 3.1 A Failure to Communicate Empathy, Not a Failure to Empathize

Pain in the media was understood in terms of the lived experiences of participants; that is, adolescents identified with popular media and could empathize with and place themselves in the shoes of various media characters. Adolescents discussed the avoidance of pain by observers when AWLEP were in pain, likening it to their own lived experiences

I feel like there's a lot of like talking around the pain, specifically. Because things like that [pain] can be really difficult to talk about in like a genuine way. So instead it would be talking about other seemingly unrelated things or just, you know, bickering. (Rory, 18 years old, genderqueer, does not experience chronic pain)

Pain was viewed by adolescents as being the ‘elephant in the room’ and was often not directly addressed, but rather pain was danced around and avoided. Adolescents noted that they, like media characters, would engage the AWLEP in ways that disconnected them from their pain experience. As observers, this allowed them to avoid their own as well as the AWLEP's discomfort. Avoidance of addressing AWLEP pain could be interpreted as a lack of empathy for AWLEP. However, Rory noted that they lacked the language necessary to address and validate the AWLEP's pain experience. For them, distraction was their only option.

Sometimes you don't have the words to say ‘hey, I'm really sorry, this sucks and I don't know how to help you’. So instead, it'll just be like ‘ok well how can I distract this person?’ (Rory, 18 years old, genderqueer, does not experience chronic pain)

Rory provides a compassionate view of observers who appear to display a lack of empathy for AWLEP's pain. Adolescents feel empathy for AWLEP, yet they perceive that they do not have the language and tools necessary to communicate empathy for the AWLEP's pain and distress. However, it may be that the issue is a lack of confidence to use the language and tools at their disposal for addressing AWLEP pain. In the above quotation, Rory provides an example of a validating statement (i.e., ‘hey, I'm really sorry, this sucks and I don't know how to help you’) but notes that it is easier to distract the AWLEP. Despite having the language at their disposal, adolescents may avoid addressing AWLEP pain, either to avoid distress, discomfort in communicating empathy, or a lack of confidence necessary to validate AWLEP pain. Rory reflects further on avoiding AWLEP pain and suggests that observers may seek to avoid making things worse for the AWLEP.

How can I just avoid this topic altogether instead of bringing it up and making it potentially more painful or saying the wrong thing? It just tends to be a bit easier that way. (Rory, 18 years old, genderqueer, does not experience chronic pain)

Observers in the media may be failing to communicate their empathy by validating AWLEP for fear of making things worse or more painful for the AWLEP. Observers may also fail to communicate their empathy towards AWLEP to avoid their own distress associated with the AWLEP's pain experience. Another belief that may underpin this failure to communicate empathy, is a belief that it will be unwanted by the AWLEP. Leon explains:

In the… clip at the abortion clinic when the guy who did care actually tried to care for the woman, she pushed him off, right? Because some people, when they're in pain, they're in denial of help and they don't like to rely on others. … A lot of people think that they're supposed to not be weak around others because crying is a sign of weakness, or showing that you're in pain is a sign of weakness, because no one wants to accept the fact that other people might actually care, not make fun of them for being in pain (Leon, 17 years old, male, experiences chronic pain).

Leon is describing that the inability to communicate empathy for a AWLEP's pain arises from a conflict within both those who observe others in pain *and* those who are experiencing pain. Importantly, he refers to some people who are in pain as being in ‘denial’ of help. The choice of words here is striking. Denial implies that the AWLEP is refusing, or would refuse, something that they desire or need. The very word implies that the AWLEP is in conflict with themselves, and they are avoiding the thing that they actually need: empathy and validation of their pain from another. Leon is also describing a culture of pain that is deeply rooted in stoicism; that our society promotes and reinforces AWLEP concealing and internalizing their pain in order to not appear ‘weak’. He goes further to attribute this failure to communicate empathy, as a reflection of the AWLEP's failure to accept the *fact* that others do care about their pain. Leon is communicating that caring for another's pain is the truth; the communication breakdown is deeply rooted in a society and culture that shapes a stoic AWLEP who can only expect to be taunted, mocked, and even criticized because of their pain.

Despite these reasons and beliefs that may lead to a failure to communicate empathy, Kennedy (who has lived experience with chronic pain) suggests that a AWLEP's failure to see empathy being communicated by observers in the media can and should be addressed. She goes further to offer specific recommendations about how this could be resolved.

Um so I think it's like important for proper representation of like you know like ‘try this instead’ or like the friend being supportive and like, you know, trying to get her to talk about her pain or help her with her pain rather than try and forget about it. (Kennedy, 17 years old, female, experiences chronic pain)

Kennedy deliberately uses the word ‘proper’ to describe how observers *should* react to AWLEP pain, highlighting what she considers to be ‘good practice’. She elaborates by exploring the idea that ‘proper’ representation would include helping the AWLEP and offering coping mechanisms, rather than pushing the AWLEP to forget about their pain. Participants noted that comprehensively representing and communicating empathy has important implications.

Because in the end, like it's a type of coping mechanism that will like destroy you more than help you. (Kennedy, 17 years old, female, experiences chronic pain)

Kennedy explores the idea that by pushing AWLEP to forget about their pain rather than recalling, addressing, and validating it, we are ‘destroying’ the AWLEP. This idea highlights the importance of the social context of pain. Although observers may be wary of making things worse for the AWLEP, avoiding pain leaves AWLEP to experience their pain alone. Without affirmation/validation within a community, AWLEP are isolated with pain that lingers and is unaddressed. Kennedy highlights that pain is not experienced alone, and we do not get better alone; rather, empathetic observer responses promote adaptive coping.

The theme ‘a failure to communicate empathy, not a failure to empathize’ provides a compassionate view of observers. Adolescents experience empathy for AWLEP pain but lack the tools and confidence to communicate their empathy. An observer's lack of confidence may be associated with an attempt to avoid making things worse for the AWLEP or distancing themselves from their own distress. It could also be a lack of knowledge about what is and is not validating and helpful. However, a failure to communicate empathy in the media can and should be addressed. Participants with chronic pain identified that avoiding talking about pain and pushing AWLEP to forget about their pain left them to experience pain in isolation and could have consequences. Empathetic observer responses promote adaptive coping and highlight the social context of pain.

### 3.2 Media as the Metric

A salient issue among participants centered around the power of media, with adolescents identifying how the media impacts their attitudes, beliefs, and perceptions. This was particularly true in the context of how pain should be experienced and when that experience should be believed by others. Participants noted that media depictions of pain are frequently internalized and establish norms about how pain should be experienced. Uniquely expressed by participants with chronic pain, some felt as though if they did not express their pain in the manner depicted by the media, observers would not believe them. This theme is described by participants' reflections on the power of media to define cultural norms about pain. The way media socializes us to understand pain, in turn, influences whose pain is empathized with and why.

Like I don't know, it was just setting this standard that like if I'm not making a big deal about it or I'm not like ‘ah, like my stomach hurts’ like it doesn't. Like if I'm not saying it, it doesn't hurt, but it does. It's just that I'm not vocalizing it. (Kennedy, 17 years old, female, experiences chronic pain)

This speaks to the idea that a social hierarchy could be embedded within the social context of the pain experience and is reinforced by the media. The media appears to hold greater power to create norms around the pain experience than individuals' real‐world, lived experiences and thus plays a dominant role in shaping how pain is understood. These norms may subsequently be internalized by adolescent observers (the middle of the hierarchy) and placed upon AWLEP (the bottom of the hierarchy). If adolescents living with pain do not experience and express pain according to the norms set forth by the media, they risk having their pain overshadowed or disbelieved. Kennedy speaks to the experience of suppressing her pain, which may not be aligned with media norms around expressing pain. Nora, who has lived experience with chronic pain, shared that the media frequently depicted and reinforced distorted perceptions of pain wherein pain was only visible to the (observer's) naked eye.

But with like scoliosis, I've like watched shows where the only thing they really show about scoliosis is people who have like super severe scoliosis where they're like… you can see their physical deformities and like that's not how I really am and I still have pain. It's like, it kind of gives people the idea of like ‘Oh well, if you're… your scoliosis isn't as severe as this, then you don't need surgery, so your scoliosis isn't as bad’. (Nora, 17 years old, female, experiences chronic pain)

Participants who experienced chronic pain endorsed the belief that adolescents consuming media were internalizing the belief that for pain to be present, it must be visibly detected by others. This may reinforce the belief that if an AWLEP does not express their pain in a certain manner, then their pain is less real. Nora defends that despite not having physical deformities, she still experiences pain. Media depictions provide a metric by which we can compare pain experiences. For instance, Nora states that observers hold the belief that because she does not perform/experience pain according to the media's metric, her pain is less severe and undeserving of surgical treatments. She is further conveying that the metric set by the media, and not meeting it, could influence the medical care she receives; it could influence health care providers' decision making. Nora further noted that the nuances of the pain experience were either misrepresented or absent entirely.

They don't really like focus on like even just like the pain of not having to… to go into like operations for that [scoliosis]. (Nora, 17 years old, female, experiences chronic pain)

Adolescents with pain noticed the misrepresentations and/or lack of representation of their lived experience. Some aspects of the pain experience, such as physical deformities and surgical treatments, were captured by the media; however, other aspects of the pain experience are diminished or absent entirely. Physical deformities and treatments can be seen by others, whereas the lived experience is often deeply personal and subjective. Media could provide a metric by which we compare the pain experience of individuals. However, participants provided a powerful reminder that these pain metrics cannot be applied universally.

It's just everyone has it in a different way, and everyone's pain will be different, and they'll react to it different, and have to treat it differently. (Nora, 17 years old, female, experiences chronic pain)

Context matters, and participants with chronic pain acknowledged that pain experiences will feel different and be expressed differently for everyone. But Kira, a young participant who lives with pain, observed that to get an audience's attention, pain in characters needs to be worse, more distressing, or more emotional. She says,

if you don't have a full back story of a character, it's hard to portray pain the way like you usually would… In real life, you won't be making huge faces and like a lot of sound when you're in pain. But if you wanted to… show it in a movie and or show like, the actor sort of has to play that cause that's, that's like the way it's written in the script and it might not be like realistic, but I guess to people who don't have pain it can like bring the message across easier. (Kira, 14 years old, female, experiences chronic pain)

The degree to which you bend the message depends on the audience. Kira agrees that these portrayals of pain are exaggerated, but suggests that this is what audiences without pain need to see to understand the message. Kira's comments may also be conveying her experiences that her peers who don't live with pain don't acknowledge her real lived experiences. To get their attention, pain in characters needs to be worse, more distressing, more emotional. This could reflect the lack of validation and attention she receives in her real life and also reflects her belief that bending representation could evoke the empathy she and others need and are lacking.

The pressure to express pain according to a metric set forth by the media was uniquely expressed by participants with chronic pain. Media is highly influential in not only spreading but also creating the very messages about pain that will then be internalized by youth. These messages influence how those who experience chronic pain believe they need to communicate their pain. Additionally, popular portrayals normalize and privilege certain ways of managing or coping with pain.

I feel like for that to be in the media in like such a popular show, it just like suggests like drugs is the answer. Like I know that's like that's a pretty big statement, but like they're saying instead of like dealing with it or like I don't know, getting a first aid kit or something like, they're just like ‘whatever, I'll smoke a joint and I'll feel better’ and it's like a temporary fix and not like an overall one. (Alissa, 16 years old, female, experiences chronic pain)

Wanting a quick resolution of your pain is normal, but Alissa's concern, which is echoed by other participants, is that when popular shows don't portray alternatives, youth watching these shows will consider quick resolutions for their pain as the go‐to solution.

Miryam, who lives with chronic pain, speaks to the potential power of media to foster an understanding of their lived experiences of pain when those experiences are represented authentically.

You know they're like, ‘oh like, you can just deal with that like it's nothing big’. But when the media starts to properly represent it and show how much it really affects you, like it means a lot more, especially to those people suffering from it. Because especially if you like have a job or you're at school and people expect you to like, be doing a lot of things, it's like ‘I'm not able to, but you wouldn't see that unless you understand it’. (Miryam, 17 years old, female, experiences chronic pain)

If pain in media was ‘properly’ represented, adolescents believed it could evoke understanding and empathy for pain in the real world. Since pain may not be immediately visible to observers, media could be harnessed to build a broader, more multidimensional understanding of pain, and particularly a more accurate understanding of what it is like to *live a life with* chronic pain. Participants' observations on how media might be harnessed as a catalyst for change are discussed in the next theme.

The theme ‘media as the metric’ captures the notion that norms and standards about pain are created by the media. If AWLEP did not express their pain according to the standards set by the media, they believed that they risked having their pain disbelieved and their medical care compromised. Aspects of the pain experience were frequently misrepresented or absent entirely and impacted whether an AWLEP's pain experience was believed, including in the context of receiving adequate healthcare. The norms and standards set forth by the media were internalized by adolescents with chronic pain and provided a metric by which they could compare pain experiences. As identified by a participant without chronic pain, it is important that those with lived experience be able to contribute to the creation and conversations surrounding pain in the media.

### 3.3 Media as the Conduit for Transformation

Participants noted that the media can act as a channel for knowledge transfer and provide mass exposure to, and representation of, experiences adolescents may not otherwise encounter. Adolescents expressed that these depictions could be harnessed to build and transform empathy among consumers of popular media. This theme reflects participants' optimism that media can transform understanding and empathy. These comments serve as calls‐to‐action for media creators to improve representation and, in doing so, improve cultural attitudes towards pain and those who experience it.

Rory, who does not have chronic pain, noted that the media have the potential to broaden their emotional repertoire and ability to empathize with AWLEP, particularly when pain is depicted properly.

Cause I think that's important for building empathy too, like some people if you know, someone you don't really know that well says ‘hey, I am in a lot of pain all the time’ like, you're in a lot of pain all the time, OK? But um like unless you're like close friends with that person and you can see it on a daily basis and you can see how much it affects their life it doesn't really have an impact on you. But when you see it in a character on a screen who you're watching progress through their life and you see this thing consistently affecting them and you see how it impacts your daily life and their functioning and just how frustrating it can be. Then you can gain empathy for the real people who experience that, and it can become more important to you. It can broaden your emotional scope I guess. (Rory, 18 years old, genderqueer, does not experience chronic pain).

Again, Rory describes a social hierarchy wherein the pain experience of real‐world AWLEP, particularly of strangers, is undervalued. Thus, if real world pain experiences do not have a direct impact on observers (i.e., if the AWLEP is not personally known by them), observers can relatively lack empathy for their pain and suffering. However, Rory articulates how the media provides a conduit through which observers can learn to adopt the perspective of AWLEP. Adolescents identified feeling more connected with characters in the media than strangers in their real life, as they are exposed to the characters' everyday lives, including their pain experiences. These perspectives may be internalized and afford adolescents the ability to engage in perspective taking and translate the empathy they gain for media AWLEP to real world AWLEP.

Adolescents also expressed that when characters in media observed another character's pain and then showed and communicated empathy for them, they considered this to be a courageous and brave act: Some adolescents even considered this to be ‘heroic’. As Toby explains:

I think we do need more like heroic acts. I feel like that's something that's kind of missing in cinema altogether. But we need people to take initiative, so I feel like that's something that we lack. … Heroes were created. You know, during war times rough times, people needed something to kind of boost motivation and morale. So even with today, with some you know sexual harassment and whatnot, people getting beat altogether just need somebody to call it out for what it is. (Toby, 16 years old, male, does not experience chronic pain)

This statement highlights the potential and unharnessed potential of media portrayals of pain. It raises an important question: Why is responding with empathy framed as ‘heroic’? It could reflect the enormous societal pressure and conditioning to conceal, deny, and avoid not only our own pain, but the pain of others. Toby argues that if media consistently depicted empathetic responses as normal and valued, the effects could extend beyond the screen. By drawing on his understanding of historical wartime communication that fostered mutual support, he suggests that storytelling can mobilize collective action by convincing audiences they are capable of courageous behaviour. In watching stories where characters show compassion for others, adolescents may be inspired to respond to others' pain with greater empathy.

Not all participants agreed that media failed to cultivate empathy for those who experience pain. Kira says,

I think it depends cause a lot of the times in movies and TV shows when the character is in pain, you're made to be, to feel like sympathetic with the person who's in pain and sort of the people that are hurting the character that's in pain they're like the villain in the movie or TV show. But yeah, I think in general a lot of movies and TV shows just make you feel or like try and make you feel bad for the person who's in pain, so I don't think… I don't think people would necessarily like be mean or cold in real life because of movies, because of how much like characters in pain like you're meant to feel sympathetic with them. (Kira, 14 years old, female, experiences chronic pain)

In Kira's assessment, villains in movies and TV shows are characterized by their lack of empathy for people in pain. Kira sees this empathy or lack thereof as shorthand for establishing the protagonist and antagonist. This characterization, albeit shallow, could lay the groundwork for more complex and diverse portrayals of pain and empathy. A participant without chronic pain articulated a call to action to ensure that the lived experiences of AWLEPs are included in pain depictions and further, that this media should be created and produced by people with lived experience.

There needs to be more diversity in you know, the writers' rooms of these things. Because, you can't come at the pain of say, a Black gay man, from the perspective of a cisgender heterosexual white woman. You just can't do that. You won't have as nuanced an understanding which can lead to, you know, more exaggerated things like that. So I'd say more diversity in writers' rooms from people who have experienced that pain instead of people who are seeing it and trying to replicate it. (Rory, 18 years old, genderqueer, does not experience chronic pain)

By including screen writers who have experienced pain in the creation of popular media, we can promote the lived experience of AWLEPs in pain depictions. Rory highlights the need to engage those with chronic pain in the creation of popular media by analogizing a cisgender, heterosexual white woman writing about the pain of a Black, gay man. In both scenarios, there are misrepresented elements of the lived experience that may contribute to an inflated and/or unrealistic metric by which we compare pain experiences. And as discussed above, these misrepresentations can have real world, and serious, consequences. The theme ‘media as the conduit for transformation’ captures the power of the media, when harnessed correctly, to build empathy for pain and skills to communicate that empathy to AWLEP in the real world. Adolescents noted that they often connect more deeply with characters in popular media than with real‐world AWLEP they do not personally know, allowing them to adopt these characters' perspectives and feel a deeper sense of empathy for them. When pain is depicted effectively, this perspective taking towards media characters with pain can help adolescents build empathy that they can later translate to real‐world interactions. Further, when pain experiences are depicted accurately, adolescents are exposed to a more multidimensional understanding of living with pain, which fosters an understanding that goes beyond the physical sensations alone.

## 4 Discussion

This exploratory study examined how adolescents with varying lived experiences of pain perceive and discuss pain depictions in popular media. Reflexive thematic analysis of focus groups generated three themes: (1) ‘a failure to communicate empathy, not a failure to empathize’, (2) ‘media as the metric’, and (3) ‘media as the conduit for transformation’. Each theme considered the potential power of media in socializing adolescent pain experiences and igniting empathy.

The first theme sheds light on previous research. Cormier et al. (2024) found that most pain instances in popular adolescent media occurred in social contexts (i.e., 85% of pain events were witnessed by at least one observer), yet only 13% of observers engaged in any prosocial behaviours in response to pain. These findings portray observers as unempathetic; however, participant responses in the current study provided a compassionate view of an observer's failure to communicate and respond. Participants identified that observers may lack the language and tools, and perhaps the confidence and knowledge, to communicate their empathy effectively.

Adolescents expressed that they would distract versus validate others' pain because they believed the latter could make pain worse if they said or did the ‘wrong’ thing. Research has identified that parents express similar fears and may avoid reminiscing about painful events with their children (Pavlova et al. 2022). However, guiding conceptual models of chronic pain posit that pain avoidance is a psychological factor underlying the development and maintenance of chronic paediatric pain (Asmundson et al. 2012). Acceptance‐based therapies highlight the importance of confronting rather than suppressing chronic pain to reduce its interference (Noel et al. 2012; Wetherell et al. 2011). Validation from observers has also been shown to significantly improve AWLEP emotional well‐being, reducing worry and pain intensity (Linton et al. 2012; Leong et al. 2015).

Adolescent observers may also avoid talking about pain and fail to act empathically because observing AWLEP may cause them distress. According to Goubert et al. (2005) conceptual model of pain empathy, when observers experience self‐oriented distress in response to the pain of others, they may be more likely to turn inwards and fail to act empathically towards AWLEP. Alternatively, observers may experience other‐oriented emotions wherein they are able to separate their own distress from that of the AWLEP, thereby leading to more empathetic and adaptive responses. Whether observers engage in self‐oriented or other‐oriented empathetic responses is dependent on top‐down processes (e.g., prior personal experience with pain) (Goubert et al. 2005). Media portrayals of pain could be another source of information about pain that could influence their process of empathizing with others.

A second theme, ‘media as the metric’, provided an account of the media serving as the metric by which we compare real‐world pain experiences. Although adolescents may learn about pain through media consumption, this is primarily beneficial if pain is being adaptively represented. However, the types of pain commonly experienced by adolescents (i.e., chronic pain and medical/procedural pains) are misrepresented *and* underrepresented in adolescent popular media (Cormier et al. 2024). Pain caused by violence and injuries dominates media representations and is frequently dismissed by AWLEP and observers (Cormier et al. 2024).

Exploratory findings suggested that media may establish norms and standards for how pain should be experienced and expressed. One participant emphasized that real‐world AWLEP must explicitly communicate their pain to avoid disbelief. Prior research shows that adolescents with chronic pain often hide their pain to avoid judgement or social burden, while some choose to endure pain silently due to fears about invasive procedures or alarming family members (Sabetsarvestani et al. 2024; Wakefield et al. 2022). Disbelieved and invalidated AWLEP face consequences, including stigma from healthcare providers, peers, and family, leading to poorer physical health and emotional distress like anger, guilt, and depression (Kool et al. 2012; Newton et al. 2013). Media creators must recognize the behavioural standards they promote and their impact on adolescent AWLEP. One participant further stressed the need to engage AWLEP in the creation of accurate pain portrayals.

Narrative possibilities explore not just what is, but what could be (Beatty 2017). Depicting diverse pain narratives in media could aid in recognizing and validating pain. Participants in this study noted chronic pain was often unrepresented. One participant highlighted how media's focus on visible pain while ignoring invisible suffering contributed to scepticism about the effects of chronic pain. Expanding media's narrative scope could help adolescents understand that all pain is valid, regardless of the form it takes.

A third theme ‘media as the conduit for transformation’ described the inherent power of the media in building adaptive understandings of adolescent pain experiences. Participants noted that the representation of pain in the media powerfully influenced how pain is experienced, expressed, and responded to in the real world. Adolescents also expressed that the media could provide exposure to experiences they may not otherwise encounter. Previous research suggests that pain‐related beliefs and behaviours may be learned by observing others (Goubert et al. 2011; Boerner et al. 2017). Given that observational learning could occur through media consumption (Krahé and Möller 2010; Mullin and Linz 1995), it is possible that observational learning about pain extends to media consumption. Therefore, it is critical that the media properly represents pain depictions to promote adaptive learning about pain.

Observational learning may also extend to the development of empathy. Studies have examined whether perspective taking, a key ingredient for the development of empathy (Batson et al. 1997), can be induced by watching movies. The results of these studies have found that perspective taking and empathy could be achieved through media consumption (Ahmadzadeh et al. 2019). Adolescents in the current study noted that media consumption expanded their empathy for pain, supporting the idea that media can promote more empathetic observer responses. As discussed in the first theme, ‘a failure to communicate empathy, not a failure to empathize’, adolescents are lacking channels by which they can adopt the language, tools, and confidence to adaptively respond to pain; however, the media may provide a channel for this learning to occur. There is also evidence to suggest that empathy is directly related to acting prosocially (Underwood and Moore 1982). Consuming prosocial media may lead to an increase in prosocial behaviours, effectively increasing an observer's confidence to act empathetically (Prot et al. 2015).

Harnessing the media to develop empathy has important implications, particularly for those who experience inequities in pain care. Racialized minorities experience disparities in the recognition and treatment of pain (Green et al. 2003). However, it has been hypothesized that empathy may be an important means for reducing racial disparities in pain, and a study by Drwecki et al. (2011) found that participants who completed a perspective taking intervention to imagine how pain affects patients' lives experienced a 55% reduction in pro‐white treatment biases.

This study had limitations. First, more diverse perspectives should be included. Adolescents with chronic pain were included in only one focus group to ensure diversity in the study sample based on that aspect of lived experience, meaning that findings for this subgroup are exploratory and may not capture the full heterogeneity of experiences. Moreover, the aim of this study was not to examine group differences or generalize. Racialized and minoritized groups are also underrepresented in adolescent media (Cormier et al. 2024) and the perspectives of more diverse adolescents should be examined. In addition to adolescent perspectives, future research should examine parents, teachers, and healthcare providers. Moreover, given the potential of media to change portrayals of pain and build understanding and empathy, future research should include creators of popular adolescent media. Second, this study was based on one prior study of pain in popular adolescent media (Cormier et al. 2024). Future research should explore other types of adolescent media, including those believed to portray pain in more prosocial ways. Lastly, although adolescents reported that learning about the impacts of pain from AWLEP media characters could build empathy, the study did not explore whether this process contributed to self‐oriented distress. Examining whether adolescents experience distress in response to viewing characters in pain would strengthen understanding of how adolescents internalize media portrayals.

Pain in popular adolescent media may be internalized by adolescents and transformed to evoke empathy. The first theme, ‘a failure to communicate empathy, but not a failure to empathize’, provided a compassionate view of the unempathetic observer. Adolescents in the current study noted that they avoid talking about AWLEP's pain, which may be attributable to a lack of confidence or fear of making the pain worse. The second theme, ‘media as the metric’, highlighted the notion that the media provides the metric by which we can compare pain experiences. This has important implications for the adolescent pain experience, including stigma and disbelief, if AWLEP do not conform to the standards set by media representations. The third and final theme, ‘media as the conduit for transformation’, underscored the potential power of media. If pain was ‘properly’ portrayed, by accurately reflecting lived experience and portraying empathy, it could translate to the real world: Media could create ‘heroes’ and spark a critical and needed social movement.

## Author Contributions

A.C. wrote the initial manuscript and facilitated the focus groups with the patient partner, K.D. A.C. under the supervision of the senior author (M.N.) was responsible for overseeing the project. All authors contributed significantly to this manuscript in reviewing the results, development of the analysis, and finalizing the final manuscript.

## Funding

The authors have nothing to report.

## Ethics Statement

Our study was approved by Conjoint Faculties Research Ethics Board, University of Calgary (Ethics ID: REB21‐1308). One participant completed an assent form, accompanied by a parent who provided written informed consent, while all others gave written informed consent before enrolling in the study.

## Supporting information


**Appendix S1:** Focus group schedule.
