# Supplementary material for: ‘Heroes Were (And Could Be) Created’: An Exploration of Adolescents' Perspectives of Portrayals of Pain and Empathy in Media
Source: Eur J Pain. 2026 Jun 17;30(6):e70315. doi: 10.1002/ejp.70315 (PMC13276014; doi:10.1002/ejp.70315)
Supplement: Supplementary file 1 — Appendix S1: Focus group schedule. [file EJP-30-0-s001.docx]

**Appendix**

**Focus Group Schedule**

1. How realistic do you think the pain was?

2. What did you notice about the other people in the clips?

3. Did you notice anything about the role of gender in these clips? How about race and sexuality?

4. Was there anything else that you noticed about this clip?
